# Deletion of Sirt3 does not affect atherosclerosis but accelerates weight gain and impairs rapid metabolic adaptation in LDL receptor knockout mice: implications for cardiovascular risk factor development

**DOI:** 10.1007/s00395-013-0399-0

**Published:** 2013-12-27

**Authors:** Stephan Winnik, Daniel S. Gaul, Frédéric Preitner, Christine Lohmann, Julien Weber, Melroy X. Miranda, Yilei Liu, Lambertus J. van Tits, José María Mateos, Chad E. Brokopp, Johan Auwerx, Bernard Thorens, Thomas F. Lüscher, Christian M. Matter

**Affiliations:** 1 Division of Cardiology, Department of Medicine, University Hospital Zurich, Winterthurerstrasse 190, 8057 Zurich, Switzerland; 2 Cardiovascular Research, Institute of Physiology, University of Zurich, Zurich, Switzerland; 3 Division of Cardiology and Department of Medicine, GZO-Regional Health Centre Wetzikon, Wetzikon, Switzerland; 4 Center for Integrative Genomics, University of Lausanne, Lausanne, Switzerland; 5 Center for Microscopy and Image Analysis, University of Zurich, Zurich, Switzerland; 6 Swiss Center for Regenerative Medicine, University Hospital Zurich, Zurich, Switzerland; 7 Laboratory of Integrative Systems Physiology, School of Life Science, Ecole Polytechnique Fédérale de Lausanne, Lausanne, Switzerland; 8 Zurich Center for Integrative Human Physiology, University of Zurich, Zurich, Switzerland

**Keywords:** SIRTUIN 3, Atherosclerosis, Metabolism, Oxidative stress

## Abstract

Sirt3 is a mitochondrial NAD^+^-dependent deacetylase that governs mitochondrial metabolism and reactive oxygen species homeostasis. Sirt3 deficiency has been reported to accelerate the development of the metabolic syndrome. However, the role of Sirt3 in atherosclerosis remains enigmatic. We aimed to investigate whether Sirt3 deficiency affects atherosclerosis, plaque vulnerability, and metabolic homeostasis. Low-density lipoprotein receptor knockout (*LDLR* ^−*/*−^) and *LDLR*/*Sirt3* double-knockout (*Sirt3* ^−*/*−^ *LDLR* ^−*/*−^) mice were fed a high-cholesterol diet (1.25 % w/w) for 12 weeks. Atherosclerosis was assessed en face in thoraco-abdominal aortae and in cross sections of aortic roots. Sirt3 deletion led to hepatic mitochondrial protein hyperacetylation. Unexpectedly, though plasma malondialdehyde levels were elevated in *Sirt3*-deficient mice, Sirt3 deletion affected neither plaque burden nor features of plaque vulnerability (i.e., fibrous cap thickness and necrotic core diameter). Likewise, plaque macrophage and T cell infiltration as well as endothelial activation remained unaltered. Electron microscopy of aortic walls revealed no difference in mitochondrial microarchitecture between both groups. Interestingly, loss of Sirt3 was associated with accelerated weight gain and an impaired capacity to cope with rapid changes in nutrient supply as assessed by indirect calorimetry. Serum lipid levels and glucose tolerance were unaffected by Sirt3 deletion in *LDLR* ^−*/*−^ mice. Sirt3 deficiency does not affect atherosclerosis in *LDLR* ^−*/*−^ mice. However, Sirt3 controls systemic levels of oxidative stress, limits expedited weight gain, and allows rapid metabolic adaptation. Thus, Sirt3 may contribute to postponing cardiovascular risk factor development.

**Electronic supplementary material:**

The online version of this article (doi:10.1007/s00395-013-0399-0) contains supplementary material, which is available to authorized users.

## Introduction

Sirtuins are a family of class III histone deacetylases (HDACs) that distinguish themselves from other HDAC classes by their dependency on nicotinamide adenine dinucleotide (NAD^+^) as a cofactor [16]. Sirtuin activity is thus tightly linked to cellular energy levels, confining their activity to times of caloric restriction, the only regimen known to extend life span [12], and delay the onset of age-related diseases, including atherosclerosis and myocardial infarction [6, 21].

Among seven mammalian homologues Sirt3 is the only sirtuin that has been associated with *human* longevity and aging in health [3, 11, 30]. A distinct variability in the evolutionary conserved domain of the Sirt3 gene increased the genotype-specific survival function in a homogeneous population in southern Italy [30]. Accordingly, the presence of an intronic enhancer of the Sirt3 gene was observed to be associated with survival at old ages [3]. Finally, Sirt3 showed the highest degree of genetic variation among 24 candidate genes of aging in a population of healthy seniors aged 85 years and above, who had never been diagnosed with cancer, diabetes, cardiovascular, pulmonary or Alzheimer disease [11].

Together with Sirt4 and Sirt5, Sirt3 is targeted to the mitochondrial matrix, where—through deacetylation of a variety of substrates—it orchestrates mitochondrial oxidative metabolism and controls reactive oxygen species (ROS) homeostasis [1, 14, 23, 40]. In diverse contexts Sirt3 has been reported to regulate the tricarboxylic acid cycle and oxidative phosphorylation [1, 5, 8, 31, 33, 43]. An inevitable byproduct of these catabolic reactions is the generation of ROS. Intriguingly, Sirt3 also governs the detoxification of cellular ROS by regulating antioxidant enzymes [4, 29, 34, 36–38, 40]. In vitro and in vivo Sirt3-mediated deacetylation and activation of manganese superoxide dismutase (MnSOD) reduced cellular ROS levels [4, 29, 38]. Moreover, recent reports provide evidence for a Sirt3-dependent activation of the mitochondrial matrix protein isocitrate dehydrogenase 2 (IDH2), a major source of NADPH, which in turn is vital for the reduction of glutathione, an important cellular antioxidant [33, 43]. Sirt3-mediated activation of Foxo3a augmented the transcription of both MnSOD and catalase (Cat), thereby preventing mitochondrial ROS accumulation in cardiomyocytes [36]. Along these lines, Sirt3 has been reported to prevent detrimental oxidative-stress-related phenotypes in a plethora of settings, including cardiac hypertrophy, age-related hearing loss and ROS-induced embryonic developmental arrest [18, 33, 36].

Excess ROS, subsequent mitochondrial DNA damage and progressive respiratory chain dysfunction critically contribute to the development of atherosclerosis [13, 25–27]. Central risk factors of atherosclerosis such as hypercholesterolemia and hyperglycemia lead to mitochondrial dysfunction [20, 25]. Impaired mitochondrial integrity along with accumulating cellular ROS underlie vascular inflammation and promote atherogenesis from fatty streak formation over lesion progress to plaque rupture [26, 41]. In a recent study, Sirt3 deficiency and the accompanying mitochondrial protein hyperacetylation were reported to be associated with the development of the metabolic syndrome, a cluster of hallmark risk factors for atherosclerosis, including dyslipidemia, glucose intolerance, and central obesity [2, 10, 15]. To date, the role of Sirt3 in vascular biology and specifically in atherosclerosis remains unknown.

Sirt3 orchestrates mitochondrial metabolism and governs cellular ROS homeostasis in a plethora of disease-relevant settings. Thus, we hypothesized that Sirt3 provides atheroprotection and maintains metabolic homeostasis. We therefore investigated the role of Sirt3 in atherogenesis and energy expenditure using a loss-of-function approach in a mouse model of atherosclerosis.

## Materials and methods

More detailed information is available in the supplemental online material.

### Animals and diets

Mice were housed in cages with free access to chow and water in a temperature-controlled facility with a 12-h light/dark cycle. All experiments and animal care procedures were approved by the local veterinary authorities and carried out in accordance with our institutional guidelines. Mice with a germline *Sirt3* deletion were generated by breeding mice with floxed Sirt3 alleles (*Sirt3* ^*L2/L2*^) [7] with CMV-Cre deleter mice that expressed Cre in the male germline. Congenic C57BL6/J *Sirt3* ^−*/*−^ mice were generated through nine generations of backcrosses with C57BL6/J mice. C57BL6/J LDL receptor knockout (*Sirt3* ^+*/*+^ *LDLR* ^−*/*−^) mice (Jackson Laboratories) were crossbred with C57BL6/J *Sirt3* ^−*/*−^ mice to generate C57BL6/J LDLR*/*Sirt3 double-knockout mice (*Sirt3* ^−*/*−^ *LDLR* ^−*/*−^). Eight-week-old male *Sirt3* ^+*/*+^ *LDLR* ^−*/*−^, *Sirt3* ^−*/*−^ *LDLR* ^−*/*−^, and *wild-type* mice were fed a 1.25 % (w/w) cholesterol diet (Research Diets) or normal chow for 12 weeks and subsequently killed for fasted (unless indicated otherwise) studies.

### Assessment of atherosclerosis

En face analyses of thoraco-abdominal aortae were carried out as previously described [24, 34, 42]. Briefly, thoraco-abdominal aortae were excised and opened longitudinally. Atherosclerotic plaques were visualized by fat staining using Oil red O (ORO). In addition, plaque size and composition were analyzed in serial longitudinal cryosections of aortic roots as described [34, 35]. Fibrous cap thickness and necrotic core size were assessed by Sirius Red staining. Necrotic cores were defined as areas free of extracellular matrix between the luminal fibrous cap and the collagen-rich outer intima and/or remaining media [28, 39]. Features of plaque vulnerability were characterized by necrotic core diameter and fibrous cap thickness, each of which was averaged from three different locations per mouse (once underneath each valvular leaflet) in aortic root cross sections.

### Immunohistochemistry and immunofluorescence

Cryosections were blocked and stained using the following antibodies: anti-CD68, anti-CD3, and anti-vascular adhesion molecule-1 (VCAM-1; all Serotec). Collagen was visualized using Sirius Red.

### Electron microscopy

Mice were killed and perfused with 1 % formaldehyde and 2 % glutaraldehyde in phosphate buffer (PB; 0.1 M; pH 7.4). Aortae were explanted and aortic wall samples (rings of 1 mm length) were extracted and postfixed by immersion in the above-described fixative for 48 h followed by three washes in PB and osmication in 1 % osmium tetroxide for 1 h. After another three washes with PB, samples were dehydrated in increasing alcohol gradients up to 100 %, embedded in Epon/Araldite resin (Sigma-Aldrich) overnight and polymerized at 60 °C during 48 h. Ultrathin sections, stained with 2 % aqueous uranyl acetate and Reynolds lead citrate were imaged using a Phillips CM 100 transmission electron microscope (FEI, Eindhoven, The Netherlands) equipped with a Gatan Orius CCD camera and Digital Micrograph acquisition software (Gatan). The cellular and subcellular structure of the inner aortic wall, specifically the endothelial monolayer and form as well as microarchitecture of endothelial mitochondria were assessed and compared in a qualitative manner. One hundred mitochondria in at least three different animals per group were assessed.

### Analysis of DNA damage

Aortic DNA was extracted by ethanol precipitation using TRIzol^®^ reagent (Sigma-Aldrich). DNA lesion frequency, halting DNA polymerase progression, was assessed using long amplicon PCR as described [9, 17]. Amplification of DNA polymerase b and b-globin were assessed as surrogate for genomic DNA lesion frequency. Amplification of a 117-bp and a 10-kb fragment of mitochondrial DNA was assessed as surrogate for mitochondrial DNA damage, which is specifically susceptible to oxidative damage. The following primers were used: b-globin 5-TTG AGA CTG TGA TTG GCA ATG CCT-3′ (sense), 5-CCT TTA ATG CCC ATC CCG GAC T-3′ (anti-sense), DNA polymerase b 5-TAT CTC TCT TCC TCT TCA CTT CTC CCC TGG-3′ (sense), 5-CGT GAT GCC GCC GTT GAG GGT CTC CTG-3′ (anti-sense), 10 kb mitochondrial fragment 5-GCC AGC CTG ACC CAT AGC CAT AAT AT-3′ (sense), 5-GAG AGA TTT TAT GGG TGT AAT GCG G-3′ (anti-sense), 117 bp mitochondrial fragment 5-CCC AGC TAC TAC CAT CAT TCA AGT-3′ (sense), 5-GAT GGT TTG GGA GAT TGG TTG ATG T-3′ (anti-sense).

### Expression analyses

Aortic mRNA and protein were extracted using standard protocols. mRNA expression was analyzed by quantitative PCR using the following primers: NADP-dependent malic enzyme 5-CAG GAA CCC CCA TCT CAA C-3 (sense), 5-ACATCCTGGCTGAGGAAGC-3 (anti-sense), nicotinamide nucleotide transhydrogenase 5-GAT CCA GAT TTC CGA CTT GC-3 (sense), 5-ACT CTA CGA TGT ACT CGG CCA-3 (anti-sense), glucose-6-phosphate dehydrogenase 5-GTT GTA CCA GGG TGA TGC CT-3 (sense), 5-GCC ACC AGA TGG TAG GAT AGA-3 (anti-sense), 6-phosphogluconate dehydrogenase 5-GGGTCATCCTGCTTGTGAAG-3 (sense), 5-CATCGATGATGATGTCACCC-3 (anti-sense), isocitrate dehydrogenase 2 (IDH2) 5-CAG CAC TGA CTG TCC CCA G-3 (sense), 5-CAC CGT CCA TCT CCA CTA CC-3 (anti-sense), catalase (cat) 5′-CCC GCG GTC ATG ATA TTA AGT-3′ (sense), 5′-GAT GAA GCA GTG GAA GGA GC-3′ (anti-sense). Western blot analyses were performed according to standard protocols. The following specific antibodies were used: anti-SOD2 (Santa Cruz), anti-cat (Sigma).

### Blood analyses

Prior to harvesting mice were fasted overnight. Blood was drawn and citrate plasma was separated from corpuscular elements by centrifugation at 4 °C immediately and stored at −80 °C until analysis. Levels of interleukin 1b (IL-1b), IL-6, monocyte chemotactic protein-1 (MCP-1), and tumor necrosis factor alpha (TNFα) were determined using Bio-Plex^®^ multiplex array systems (Bio-Rad Laboratories).

Plasma lipoprotein fraction distribution was measured using a Roche-diagnostics Enzymatic kit for the Hitachi 902 robot according to the manufacturer’s instruction.

Plasma malondialdehyde (MDA) levels were determined using the colorimetric AL detect lipid peroxidation assay (Enzo Life Sciences) according to the manufacturer’s instructions. Plasma glutathione reductase activity was quantified based on the rate of NADPH oxidation using the Glutathione Reductase Assay kit (Cayman Chemical) according the manufacturer’s instructions. Isocitrate dehydrogenase 2 activity was assessed based on substrate turnover using the Isocitrate Dehydrogenase Activity Colorimetric Assay kit (Biovision) according to the manufacturer’s instructions. The NADP/NADPH ratio was measured using the NADP/NADPH Quantification kit (Biovision).

### Glucose tolerance

For glucose tolerance tests a 200 mg/mL glucose solution was prepared and injected into the intraperitoneal cavity at 2 g/kg body weight. At regular time intervals for up to 3 h following administration, blood samples were taken and glucose was measured.

### Indirect calorimetry

A 12-chamber Oxymax system (Columbus Instruments) with control of food access was used to measure oxygen consumption, carbon dioxide production, food and water intake, as well as locomotor activity in individually caged mice at 23 °C.

Mice were weighed and placed into individual metabolic cages before measurements were started. Measurements were performed for 3 days in the ad lib fed state (including 2 days of acclimatization); body weight was recorded again at the end of day 3 to rule out a loss of weight during “basal” conditions. Food access was then halted for 15 h (*Fasting*, from day 3, 5 pm through day 4, 8 am) and restored thereafter (*Refeeding*). Body weight was recorded again at the end of the experiment. Free access to water was warranted during the whole experiment. Locomotor activity (longitudinal ambulatory, “Xamb”) was assessed by infrared beam interruption.

The non-protein respiratory exchange ratio, a measurement of metabolic substrate preference, was calculated as the molar ratio of *V*co_2_ to *V*O_2_. Heat (kcal/h) = *V*O_2_ (3.815 + 1.232 *RQ*).

Individual averaged values of metabolic data (Heat, VO_2_ or respiratory quotient) for each light cycle were derived for each mouse. Group averages are presented for each genotype and light cycle.

Metabolic adaptation to *Fasting* was determined by subtracting the individual averages of *night 3* (fed) from those of *night 4* (fasted) for either heat, *V*O_2_ or the Respiratory Quotient. Metabolic rebound upon *refeeding* was assessed by subtracting the individual averages of *night 5* (refed) from those of *night 4* (fasted). Metabolic changes upon *fasting* and *refeeding* were also calculated as percent change.

### Mitochondrial acetylation, western blotting

Mitochondria were isolated from livers and gastrocnemius muscle of mice by differential centrifugation using a Mitochondrial Isolation kit (Abcam) according to the manufacturer’s instructions. Thereafter, mitochondrial protein was isolated, separated by electrophoresis and blotted using standard protocols. Membranes were probed using antibodies specific for ATPB (ATP-synthase subunit b, Abcam), acetylated lysine (AcK, Cell Signaling), and Sirt3. Antibodies recognizing murine Sirt3 were raised against the C-terminal 15-amino-acid peptide (C)DL MQR ERG KLD GQD R. The peptide was conjugated to the carrier protein KLH by the added C residue and injected into rabbits (Eurogentec). Antisera were purified using immunoaffinity chromatography.

### Statistical analysis

Metric variables were assessed for distribution using Kolmogorov–Smirnov tests. Different groups were compared using unpaired Student’s *t*, Mann–Whitney *U*, Kruskal–Wallis tests or two-way repeated measurements ANOVA with Bonferroni post hoc comparisons where appropriate. Data are displayed as interquartile ranges ± minimal/maximal values, unless indicated otherwise. Null-hypotheses were rejected at *p* < 0.05, *p* values are two-sided. Analyses were done using Graphpad Prism version 5.0d 2010.

## Results

### Sirt3 deletion does not affect atherosclerosis in *LDLR*^−*/*−^ mice

**Fig. 1:**
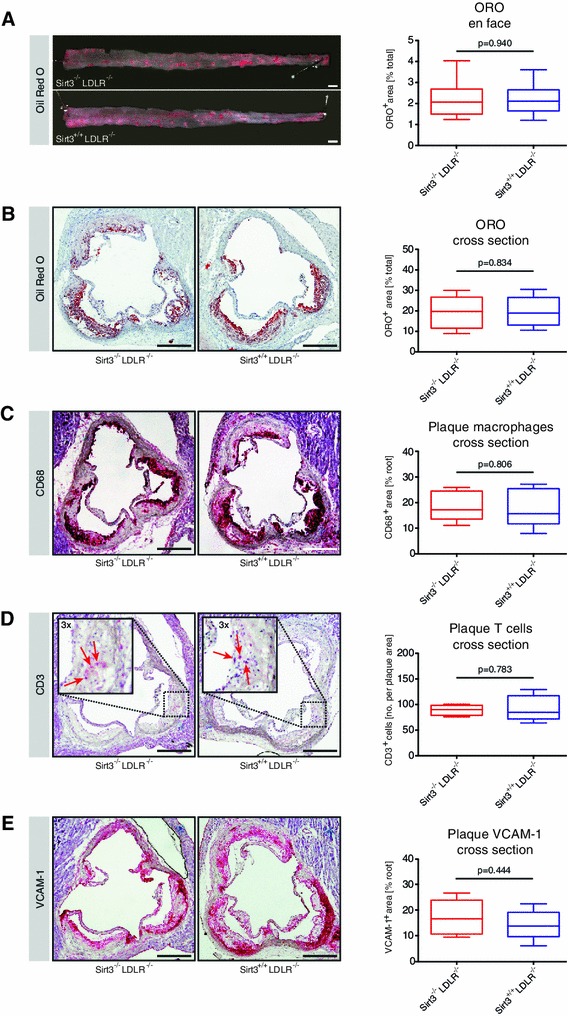
Loss of Sirt3 does not affect atherosclerosis in LDLR knockout mice after a high-cholesterol diet. 8-week old male *Sirt3* ^−*/*−^ *LDLR* ^−*/*−^ and *Sirt3* ^+*/*+^ *LDLR* ^−*/*−^ mice were fed a high-cholesterol diet (1.25 % w/w) for 12 weeks before aortae were excised. **a** Plaque burden of thoraco-abdominal aortae en face, stained with Oil Red O (ORO), *n* = 10 per group. **b–e** Cryosections of aortic roots, *n* = 6 per group, stained with Oil red O (ORO) (**b**), or immunohistochemically for CD68 **(C),** CD3 (**d**), or vascular cell adhesion molecule-1 (VCAM-1) (**e**). Images are representative micrographs, *box plots* display interquartile ranges, *whiskers* indicate minima and maxima, *scale bars* are 1 mm (**a**) and 500 μm (**b**–**e**)

After 12 weeks of high-cholesterol diet, the atherosclerotic burden of *Sirt3* ^−*/*−^ *LDLR* ^−*/*−^ and *Sirt3* ^+*/*+^ *LDLR* ^−*/*−^ mice was assessed en face in thoraco-abdominal aortae and cross sections of aortic roots. Lesion distribution and depth, as determined by ORO stainings, did not differ between the two genotypes (Fig. 1a, b).

The cellular composition of atheromata was further assessed in adjacent cross sections of the aortic root. No differences in the immunohistochemical signals for CD68 and CD3 were observed, indicating that neither macrophage nor T cell infiltration were affected by deletion of Sirt3 in this mouse model of atherosclerosis (Fig. 1c, d). To address the degree of endothelial activation, VCAM-1 expression was investigated, again without uncovering a difference between the two groups (Fig. 1e). Complementary analyses of plasma cytokine levels addressing a potential systemic inflammatory phenotype revealed no difference in IL1b, IL6, MPC-1, and TNFα between the groups (Table 1A). Similarly, plasma total cholesterol, HDL-cholesterol, LDL-cholesterol and triglyceride levels did not differ significantly between the two groups (Table 1B).

**Table 1:** Deletion of Sirt3 does not affect plasma cytokine or lipid levels

	*Sirt3* ^+*/*+^ *LDLR* ^−*/*−^	*Sirt3* ^−*/*−^ *LDLR* ^−*/*−^	*p* value
A) Plasma cytokine levels			
IL-1b (pg/ml)	99.9 ± 17.5	128.6 ± 20.6	0.317
IL-6 (pg/ml)	2.6 ± 0.3	3.6 ± 0.3	0.073
MCP-1 (pg/ml)	34.4 ± 2.1	48.9 ± 3.8	0.052
TNFα (pg/ml)	1.2 ± 0.0	1.2 ± 0.0	0.502
B) Plasma lipid levels			
Total cholesterol (mmol/l)	30.6 ± 5.0	26.2 ± 1.6	0.421
LDL-cholesterol (mmol/l)	20.9 ± 1.8	25.4 ± 4.6	0.385
HDL-cholesterol (mmol/l)	4.7 ± 0.4	5.1 ± 0.2	0.331
Triglycerides (mmol/l)	2.60 ± 0.33	2.11 ± 0.19	0.202

### Loss of Sirt3 does not alter key features of plaque vulnerability

**Fig. 2:**
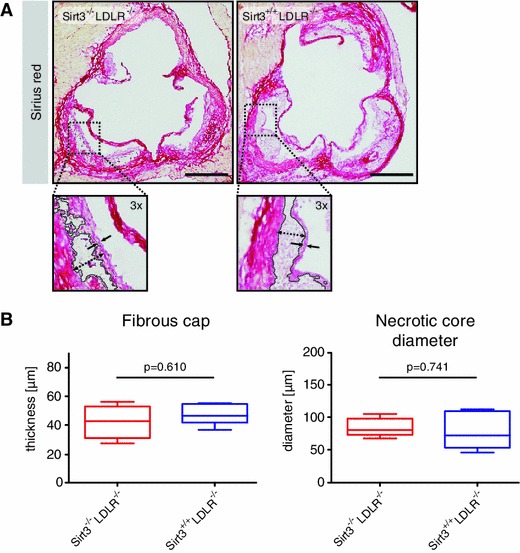
Deletion of Sirt3 does not affect features of plaque stability. *Sirt3* ^−*/*−^ *LDLR* ^−*/*−^ and *Sirt3* ^+*/*+^ *LDLR* ^−*/*−^ mice were treated as described and aortae excised. **a** Cryosections of aortic roots, stained for collagen with Sirius red, *n* = 6 per group. **b** Quantification of fibrous cap thickness, necrotic core diameter and area. Images are representative micrographs, *box plots* display interquartile ranges, *whiskers* indicate minima and maxima, *scale bars* are 500 μm

In order to evaluate potential effects on features of plaque vulnerability, we compared fibrous cap thickness and necrotic core diameter in cross sections of the aortic roots stained for collagen. Both fibrous cap thickness and necrotic core diameter did not differ between *Sirt3* ^−*/*−^ *LDLR* ^−*/*−^ and *Sirt3* ^+*/*+^ *LDLR* ^−*/*−^ mice (Fig. 2a, b).

**Fig. 3:**
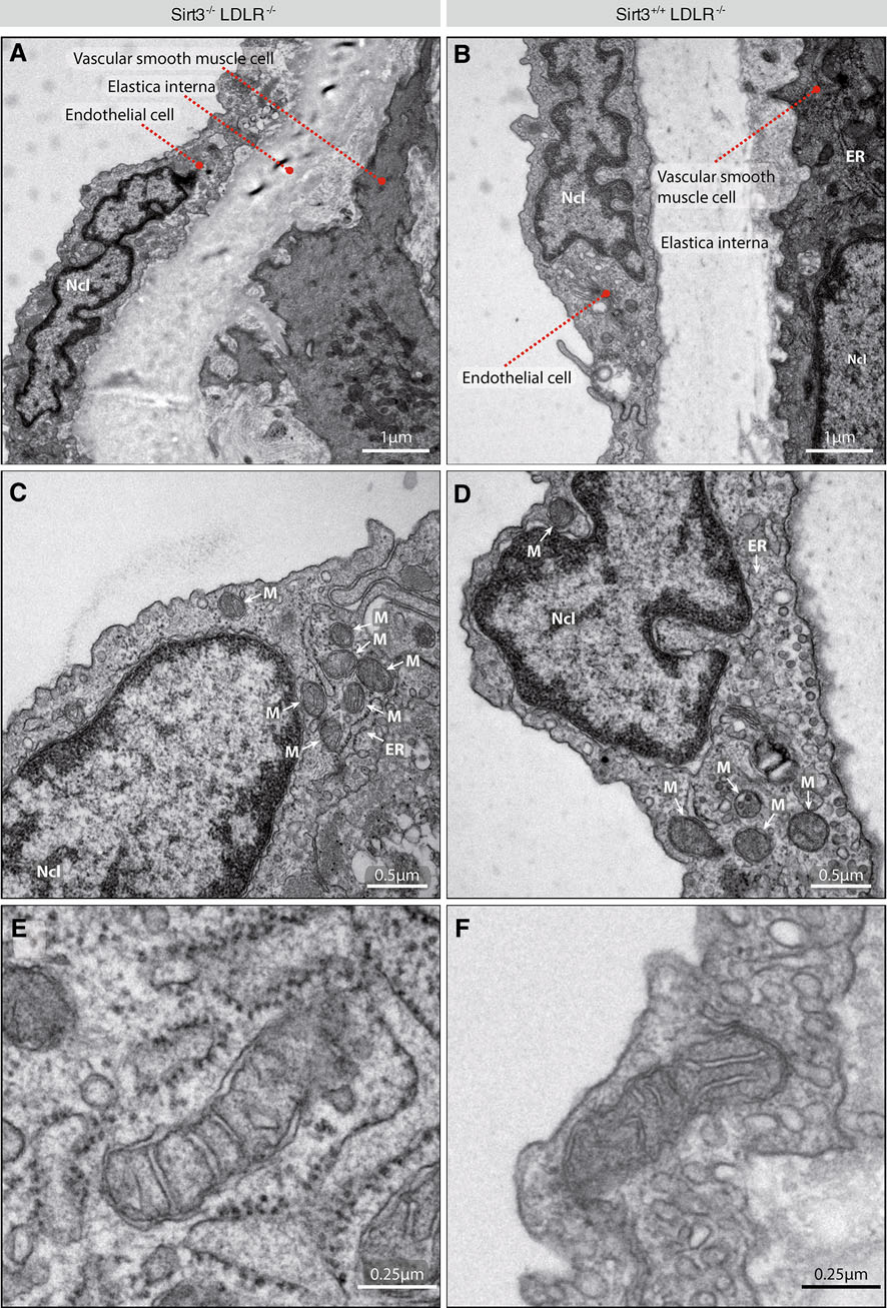
Sirt3 deficiency does not affect endothelial mitochondrial architecture in the aortic wall. *Sirt3* ^−*/*−^ *LDLR* ^−*/*−^ and *Sirt3* ^+*/*+^ *LDLR* ^−*/*−^ mice were treated as described and aortae excised and cross sections of the thoracic descending aorta were imaged using electron microscopy. **a**, **b** Overview of the inner vascular wall (from *left* to *right*): endothelial monolayer with endothelial nuclei (Ncl) protruding towards the lumen, elastica interna, vascular smooth muscle cell layer. **c**, **d** Magnification of an endothelial cell, allowing the differentiation of its subcellular components, including mitochondria (M). **e**, **f** Magnification of a single endothelial mitochondrion, showing mitochondrial microarchitecture. Images are representative micrographs of *n* = 5 per group and 100 mitochondria per aortic phenotype, and serve for qualitative comparisons only. *M* mitochondrion, *Ncl* nucleus, *ER* endoplasmic reticulum

With Sirt3 orchestrating mitochondrial metabolism, we also addressed endothelial mitochondrial microarchitecture in the aortic wall using electron microscopy. A qualitative comparison of mitochondrial number and microarchitecture revealed no Sirt3-dependent difference (Fig. 3a–f).

### Sirt3 deficiency is associated with increased levels of systemic but not aortic oxidative stress

**Fig. 4:**
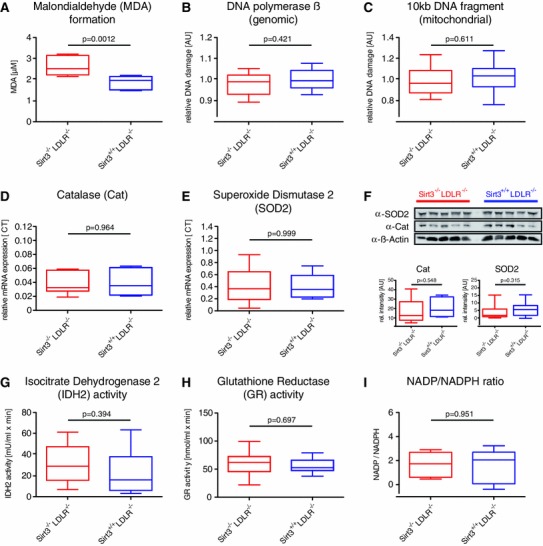
Loss of Sirt3 increases systemic oxidative stress without affecting vascular oxidative DNA damage. *Sirt3* ^−*/*−^ *LDLR* ^−*/*−^ and *Sirt3* ^+*/*+^ *LDLR* ^−*/*−^ mice were treated as described, blood was drawn and aortas were explanted. **a** Malondialdehyde (MDA) levels as surrogate for systemic oxidative stress, *n* = 8 per group. **b** Aortic DNA was isolated and relative oxidative damage of genomic (**b**) and mitochondrial DNA **c** was assessed using quantitative PCR. **b** Lesion frequency and the resulting copy number of DNA polymerase b as surrogate for *genomic* DNA damage, *n* = 10 per group. **c** Lesion frequency and the resulting copy number of a 1 kb mitochondrial DNA fragment as surrogate for *mitochondrial* DNA damage, *n* = 10 per group. **d–f** Expression of catalase (cat) and superoxide dismutase 2 (SOD2) were assessed using quantitative PCR, *n* = 9 per group (**d**, **e**) and by western blot, *n* = 5 per group (**f**). **g** Plasma isocitrate dehydrogenase 2 (IDH2) activity, *n* = 9 per group. **h** Plasma glutathione reductase (GR) activity, *n* = 6 per group. **i** Plasma NADP/NADPH ratio, *n* = 5 per group. *Box plots* display interquartile ranges, *whiskers* indicate minima and maxima

To assess putative effects of Sirt3 on systemic oxidative stress in this mouse model, plasma malondialdehyde (MDA) levels were compared. Interestingly, MDA levels in *Sirt3* ^−*/*−^ *LDLR* ^−*/*−^ mice were elevated compared with controls (Fig. 4a). Yet, a comparison of oxidative damage to the target tissue, the aortic wall, showed no Sirt3-dependent difference. Both aortic genomic and mitochondrial DNA lesion frequencies, as surrogate for oxidative DNA damage, did not differ between *Sirt3* ^−*/*−^ *LDLR* ^−*/*−^ mice and *LDLR* ^−*/*−^ controls (Fig. 4b, c and Fig S1). Accordingly, aortic expression of the antioxidant enzymes superoxide dismutase 2 (SOD2) and catalase (cat) were unaffected by the lack of Sirt3 (Fig. 4d–f). Moreover, aortic expression levels of the key NADPH-producing or -regenerating enzymes, including isocitrate dehydrogenase 2 (IDH2) and malic enzyme, were unaltered between the two groups (Fig S2). Interestingly, also systemic activity levels of IDH2 and glutathione reductase (GR) as well as plasma ratios of NADP/NADPH did not differ between the two groups (Fig. 4g–i). These findings indicate that depressed glutathione regeneration was not responsible for the observed increase in systemic oxidative stress in Sirt3^−/−^ mice. In absence of an antioxidative effect within the vessel wall, and given the lack of an effect of Sirt3 deletion on atherosclerosis, the increase in systemic oxidative stress appears unlikely to affect vascular inflammation and atherosclerotic burden in the context of advanced atherosclerosis.

**Fig. 5:**
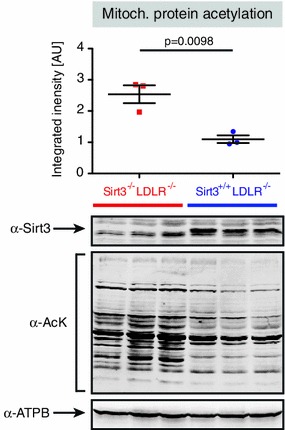
Loss of Sirt3 lead to global mitochondrial hyperacetylation. Hepatic mitochondria were isolated and protein was extracted, separated by electrophoresis and probed for Sirt3 (α-Sirt3), acetylated lysine residues (α-AcK) and the beta-subunit of ATP-Synthase (α-ATPB, loading control). Data are mean ± SEMs with superimposition of individual data points

In order to confirm a difference in Sirt3 activity in the current model (*LDLR* ^−*/*−^ mice on a high-cholesterol diet), we analyzed global mitochondrial protein acetylation in isolated hepatic and skeletal muscle mitochondria. Sirt3 deletion was associated with a marked mitochondrial protein hyperacetylation, suggesting a relevant difference in Sirt3 activity (Fig. 5, Fig S3C). To further exclude a diet- or LDLR-dependent blunting of Sirt3 activity, hepatic mitochondrial acetylation was assessed in *Sirt3* ^−*/*−^ *LDLR* ^+*/*+^ and *Sirt3* ^+*/*+^ *LDLR* ^+*/*+^ mice fed a high-cholesterol diet or normal chow. Mitochondrial hyperacetylation in absence of Sirt3 compared with *Sirt3* ^+*/*+^ controls was similar in all settings (Fig S3).

### Body weight and plasma glucose levels are increased in absence of Sirt3

**Fig. 6:**
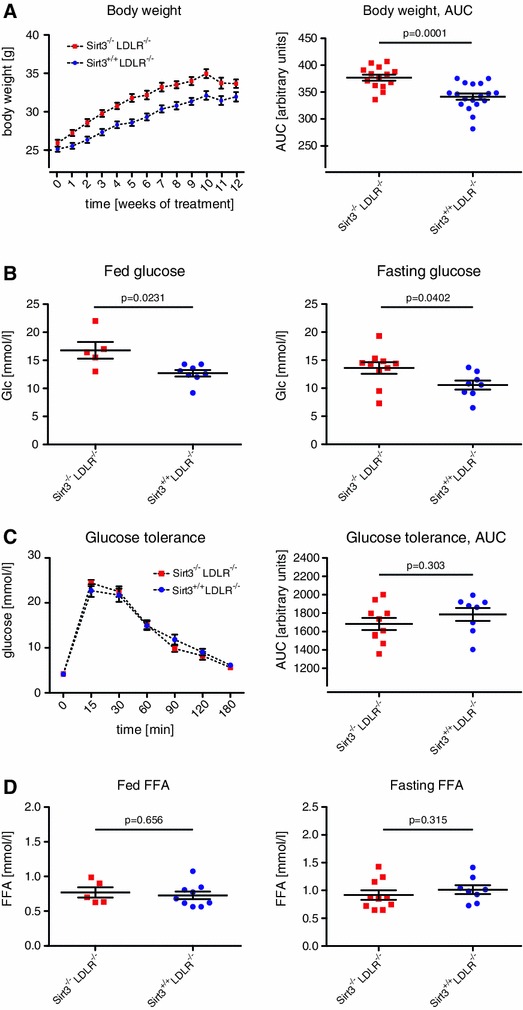
Deletion of Sirt3 accelerates weight gain and increases plasma glucose levels*. Sirt3* ^−*/*−^ *LDLR* ^−*/*−^ and *Sirt3* ^+*/*+^ *LDLR* ^−*/*−^ mice were fed a high-cholesterol diet (1.25 % w/w) for 12 weeks. **a** Weight gain during treatment (*left panel*) and its quantification comparing areas under the curve (AUC, *right panel*). **b** Plasma glucose levels, fed (*left panel*) and fasted (*right panel*). **c** Plasma glucose levels upon intraperitoneal glucose challenge (2 g/kg body weight) (*left panel*), quantification comparing areas under the curve (AUC, *right panel*). **d** Plasma free fatty acid (FFA) content, fed (*left panel*) and fasted (*right panel*). Data are means ± SEMs with superimposition of individual data points in all panels except for **a** and **c**, *left panels*

*Sirt3* ^−*/*−^ *LDLR* ^−*/*−^ mice were consistently heavier than controls (Fig. 6a). Moreover, both fed and fasted blood glucose levels were increased in absence of Sirt3 (Fig. 6b). To address whether this difference was due to a restricted capacity to metabolize glucose, we compared glucose clearance after an intraperitoneal glucose challenge. Interestingly, loss of Sirt3 did not affect glucose clearance (Fig. 6c). Moreover, plasma free fatty acid levels did not differ between *Sirt3* ^−*/*−^ *LDLR* ^−*/*−^ and control mice (Fig. 6d).

In order to rule out that the difference in body weight was related to an organ-specific phenotype or organomegaly, liver, spleen and epididymal white adipose tissue weights (WAT) were assessed. No difference between the two genotypes was observed (Fig S4).

### Loss of Sirt3 impairs metabolic adaptation to rapid changes in energy supply

To further investigate the metabolic phenotype of *Sirt3* ^−*/*−^ *LDLR* ^−*/*−^ mice and *Sirt3* ^+*/*+^ *LDLR* ^−*/*−^ controls, we compared their metabolic rates, oxygen consumption along with locomotion and food intake continuously during a period of 5 light cycles including a 15-h fasting period beginning at the end of day 3 (D3).

**Fig. 7:**
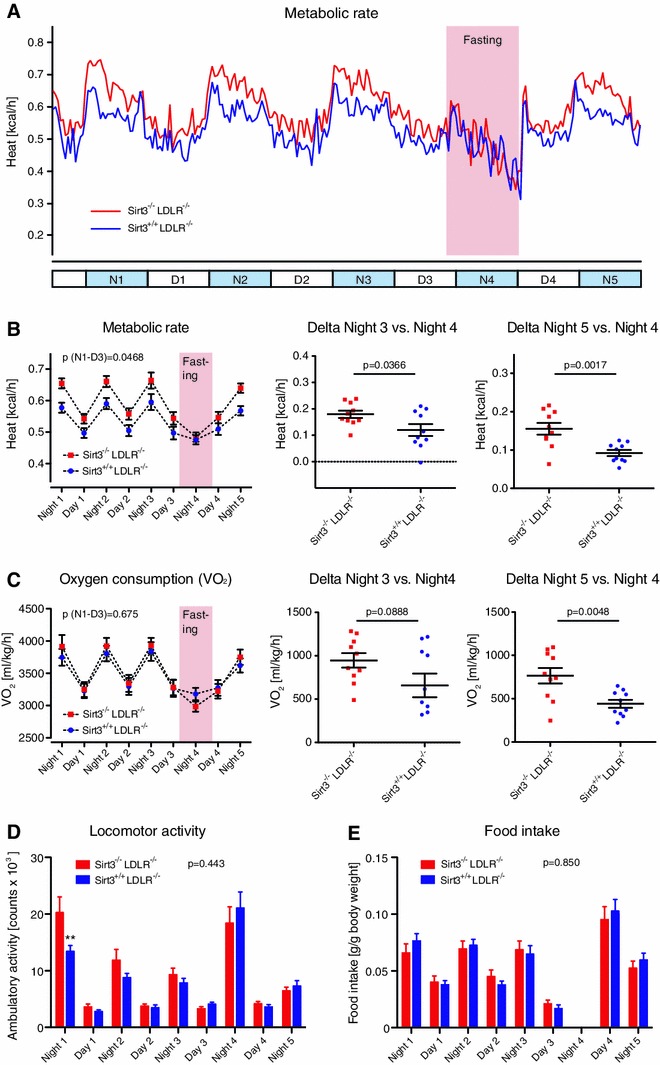
Loss of Sirt3 impairs metabolic adaptation to rapid changes in energy supply. After a 12-week high-cholesterol diet (1.25 % w/w) different metabolic parameters were assessed in individually caged *Sirt3* ^−*/*−^ *LDLR* ^−*/*−^ and *Sirt3* ^+*/*+^ *LDLR* ^−*/*−^ mice during five light cycles. **a**, **b** Metabolic rate (heat production): circadian profile during an ad libitum fed state (night 1 to day 3), during a 15-h overnight fasting period (5 pm, day 3 through 8 am, day 4) and during subsequent refeeding (day 4, night 5), *N* night, *D* day. **b** Average metabolic rates per day/night (*left panel*); metabolic drop during fasting (“delta night 3 vs. night 4”, *center panel*), and metabolic rebound upon refeeding (“delta night 4 vs. night 5”, *right panel*). **c** Average oxygen consumption (*V*O_2_) per day/night (*left panel*); *V*O_2_ drop during fasting (“delta night 3 vs. night 4”, *center panel*), and *V*O_2_ rebound upon refeeding (“delta night 4 vs. night 5”, right panel). **d** Average locomotor activity per day/night. **e** Average food intake per day/night. Data are means ± SEM, with superimposition of individual data points in “delta” *panels*. *)* p* < 0.01 compared with *LDLR* ^−*/*−^ *Sirt3* ^−*/*−^ mice

During the ad libitum (ad lib) period [night 1 (N1) to D3] *Sirt3* ^−*/*−^ *LDLR* ^−*/*−^ mice showed a higher metabolic rate compared with controls (Fig. 7a, b left panel). The fasting-induced heat-drop was more pronounced in *Sirt3* ^−*/*−^ *LDLR* ^−*/*−^ mice compared with controls (27 vs. 20 %), blunting the pre-existing difference to controls (Fig. 7a, b). Importantly, oxygen consumption (VO_2_), taking individual body weight into account, did not differ between the two genotypes during the ad lib period (Fig. 7c, left panel). However, the fasting-induced *V*O_2_-drop was also more pronounced in *Sirt3* ^−*/*−^ *LDLR* ^−*/*−^ mice compared with *Sirt3* ^+*/*+^ *LDLR* ^−*/*−^ controls (24 vs. 17 %; Fig. 7c, right panels). Thus, depletion of Sirt3 was associated with an exaggerated fasting-induced hypo-metabolism compared with controls. This exaggerated hypo-metabolism upon fasting in absence of Sirt3 could not be explained by an inability to rely on lipid utilization during fasting since respiratory exchange ratios (RER) dropped to similarly low values in *Sirt3* ^−*/*−^ *LDLR* ^−*/*−^ mice and *Sirt3* ^+*/*+^ *LDLR* ^−*/*−^ controls (Fig S5A). Whereas cumulative food intake differed between the two groups, there was no difference when food intake was normalized to body weight (Fig S5B). The experiment itself had no effect on the body weight of the animals (Fig S5C).

During *refeeding* the metabolic rebound from the fasted state was nearly twice as pronounced in *Sirt3* ^−*/*−^ *LDLR* ^−*/*−^ mice compared with controls, with heat rebound of 32 vs. 19 % in the control group and *V*O_2_ rebound of 26 vs. 14 % in controls (Fig. 7a–c). *Refeeding* behavior in terms of body weight-adjusted food intake and locomotion did not differ between the two groups (Fig. 7d, e, Fig S5B), indicating a *metabolic* rather than a behavioral phenotype.

## Discussion

### Principle findings

We demonstrate for the first time that constitutive Sirt3 deletion, despite increased systemic levels of oxidative stress, neither affects the atherosclerotic burden nor features of plaque stability in *LDLR* ^−*/*−^ mice. On the other hand, Sirt3 deficiency led to a slight increase in fasting glucose levels, whereas glucose tolerance and plasma lipid levels remained unaltered. Yet, Sirt3 deletion was associated with an accelerated weight gain and an impaired capacity to cope with rapid changes in nutrient supply, showing an exaggerated fasting-induced hypo-metabolism. Similar respiratory exchange ratios rule out a Sirt3-dependent inability to rely on lipid metabolism during fasting in the applied mouse model.

### Added value

To date no report on the role of Sirt3 in vascular diseases exists. With Sirt3 regulating mitochondrial oxidative metabolism and governing several ROS detoxifying systems [1, 14, 36, 38, 43], we postulated an atheroprotective role of Sirt3. Unexpectedly, Sirt3 deletion had neither relevant effects on the atherosclerotic burden nor on plaque stability. It can be speculated whether the lack of an atherosclerotic phenotype may be explained by model-associated specificities: the NAD^+^-dependence of sirtuins, confining their maximal activity to times of energy deprivation, may interfere with the atherogenic high-cholesterol diet applied in this study; i.e., Sirt3 activity in the control group may have been decreased by this high-caloric diet. However, the marked mitochondrial protein hyperacetylation in *Sirt3* ^−*/*−^ *LDLR* ^−*/*−^ compared with *Sirt3* ^+*/*+^ *LDLR* ^−*/*−^ mice implies a relevant difference in Sirt3 activity between the groups investigated. Although currently only indirect evidence suggesting a role for Sirt3 in cellular lipid uptake exists [19, 32], an intact cholesterol uptake and metabolism system may be required for certain Sirt3-mediated effects. Thus, the absence of the LDL receptor may interfere with distinct downstream effects.

In line with previous reports that assign Sirt3 a protective role in diverse settings of oxidative damage [4, 18, 22, 33, 36], we observed increased systemic MDA levels in Sirt3-deficient hyperlipidemic mice compared with controls. In contrast to Someya et al. [32], who report on a Sirt3-dependent glutathione-mediated oxidative detoxification, glutathione reductase activity levels did not differ in the current study, suggesting other antioxidant mechanisms such as MnSOD and catalase [4, 29, 36] to be responsible for the Sirt3-mediated antioxidant protection.

Hirschey et al. [15] recently reported that Sirt3 deletion accelerated the development of the metabolic syndrome in mice when fed a high-fat diet. *Sirt3* ^−*/*−^ mice developed accelerated obesity, insulin resistance, and hyperlipidemia. In parallel, loss of Sirt3 was associated with accelerated weight gain and elevated plasma glucose in *LDLR* ^−*/*−^ mice in the current study; however, without affecting serum lipid levels or glucose tolerance, thus not fulfilling the criteria for a full-grown metabolic syndrome [2]. Detailed assessment of this metabolic phenotype showed an impaired capacity of *Sirt3*-deficient mice to cope with rapid changes in nutrient supply. Although driving not only glucose and amino acid catabolism, but also fatty acid oxidation in times of energy deprivation [14], similar respiratory exchange ratios both during fed ad libitum and fasting periods rule out an impaired fatty acid utilization in absence of Sirt3. Interestingly, neither muscle- nor liver-specific loss of Sirt3 did manifest any metabolic phenotype under either chow or high-fat diet [7], indicating that either another organ or the interplay between different organs are necessary for Sirt3 to exert its metabolic functions in its entirety.

### Potential limitations

This study has to be interpreted in light of the following limitations: experiments have been carried out in an *LDLR* ^−*/*−^ model using a high-cholesterol diet. Therefore, a potential interplay between Sirt3 and the LDL receptor in wild-type mice under similar conditions cannot be excluded. However, to date no evidence for an LDLR-mediated role of Sirt3 exists. Moreover, a putative effect of Sirt3 on early atherosclerosis or vascular function was not assessed. Given the increased MDA levels in *Sirt3*-deficient mice Sirt3 may affect endothelial relaxation, thereby affecting the initial disposition for atherogenesis. In addition, a diet-induced blunting of Sirt3 activity cannot be ruled out completely. However, a persistent mitochondrial hyperacetylation in *Sirt3*-deficient mice, irrespective of the diet supplied or LDLR expression strongly suggests no relevant diet- or LDLR-dependent blunting of Sirt3 activity. Moreover, the electron microscopy-based analyses of the inner aortic wall and endothelial subcellular structures are qualitative in nature; no quantitative statements can be made. Finally, the exact mechanisms underlying the constrained ability of *Sirt3*-deficient mice to cope with rapid changes in nutrient supply and the causes of the accelerated weight gain remain to be determined.

### Implications

The current study provides a first step in unraveling the role of Sirt3, a key enzyme in metabolic regulation and ROS homeostasis, in vascular disease. Surprisingly, no effects on advanced atherosclerotic lesions were observed, even though levels of systemic oxidative stress were increased in absence of Sirt3. A striking acceleration in weight gain and an impaired capacity to react to rapid changes in nutrient supply underline the importance of Sirt3 in energy homeostasis. The latter findings assign Sirt3 a potential role in the development of cardiovascular risk factors, postponing the onset of distinct metabolic risk factors.

Further studies will be needed to determine the role of Sirt3 on vascular function; a reassessment of putative effects on atherosclerosis using gain-of-function studies, in the absence of a high-caloric diet, and an extended analysis of the metabolic phenotype of Sirt3 on a wild-type background and chow diet will shed further light on the putative protective roles of Sirt3 in vascular health and disease.

## Electronic supplementary material

Below is the link to the electronic supplementary material.

Supplementary material 1 (DOCX 372 kb)

## Abbreviations

HDAC: Histone deacetylase
NAD^+^: Nicotinamide adenine dinucleotide
NADPH: Nicotinamide adenine dinucleotide phosphate
ROS: Reactive oxygen species
TCA cycle: Tricarboxylic acid cycle
MnSOD: Manganese superoxide dismutase
Cat: Catalase
IDH2: Isocitrate dehydrogenase 2
ORO: Oil red O
VCAM-1: Vascular adhesion molecule-1
LDLR: Low-density lipoprotein receptor
MCP-1: Monocyte chemoattractant protein-1
IL-1b: Interleukin 1 beta
TNFα: Tumor necrosis factor alpha
MDA: Malondialdehyde
Ad lib: Ad libitum
WAT: White adipose tissue
RER: Respiratory exchange ratio
