# Supplementary material for: Deletion of Sirt3 does not affect atherosclerosis but accelerates weight gain and impairs rapid metabolic adaptation in LDL receptor knockout mice: implications for cardiovascular risk factor development
Source: Basic Res Cardiol. 2013 Dec 27;109(1):399. doi: 10.1007/s00395-013-0399-0 (PMC3898152; doi:10.1007/s00395-013-0399-0)
Supplement: Supplementary file 1 — Supplementary material 1 (DOCX 372 kb) [file 395_2013_399_MOESM1_ESM.docx]

**Deletion of Sirt3 does not affect atherosclerosis but accelerates weight gain and impairs rapid metabolic adaptation in LDL receptor knockout mice – Implications for cardiovascular risk factor development**

Stephan Winnik^1, 2^, Daniel S. Gaul^1^, Frédéric Preitner^3^, Christine Lohmann^1^, Julien Weber^1^, Melroy X. Miranda^1,6^, Yilei Liu^1^, Lambertus J. van Tits^1^, José María Mateos^4^, Chad E. Brokopp^5^, Johan Auwerx^6^, Bernard Thorens^3^, Thomas F. Lüscher^1,7^, and Christian M. Matter^1,7^

1. *Division of Cardiology, Dept. of Medicine, University Hospital Zurich, Zurich, Switzerland and Cardiovascular Research, Institute of Physiology, University of Zurich, Zurich, Switzerland*
2. *Division of Cardiology and Department of Medicine, GZO – Regional Health Centre Wetzikon, Wetzikon, Switzerland*
3. *Center for Integrative Genomics, University of Lausanne, Lausanne, Switzerland*
4. *Center for Microscopy and Image Analysis, University of Zurich, Zurich, Switzerland*
5. Swiss Center for Regenerative Medicine, University Hospital Zurich, Zurich, Switzerland;
6. *Laboratory of Integrative Systems Physiology, School of Life Science, Ecole Polytechnique Fédérale de Lausanne, Lausanne, Switzerland*
7. *Zurich Center for Integrative Human Physiology, University of Zurich, Zurich, Switzerland*

Running title: Sirt3 in Atherosclerosis & Metabolism

**Key words:** Sirtuins; Sirtuin 3; atherosclerosis; metabolism; oxidative stress

**SUPPLEMENTAL MATERIAL**

**Figure S1: Loss of Sirt3 does not increase aortic oxidative DNA damage.**

**
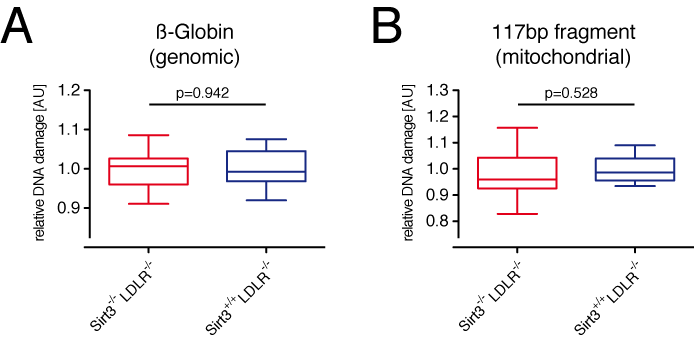
**

**Figure S1:** Eight-week old male *LDLR^-/-^* and *LDLR^-/-^Sirt3^-/-^* mice were fed a high-cholesterol diet (1.25% w/w) for 12 weeks before aortae were harvested. Aortic DNA was isolated and relative oxidative damage of genomic (A) and mitochondrial DNA **(**B) was assessed using quantitative PCR. **(A)** Lesion frequency and the resulting copy number of ß-Globin served as surrogate for *genomic* DNA damage. **(B)** Lesion frequency and the resulting copy number of a 117bp mitochondrial DNA fragment served as surrogate for *mitochondrial* DNA damage. Box plots show interquartile ranges, whiskers indicate minima and maxima.

**Figure S2: Loss of Sirt3 does not affect aortic expression levels of major NADPH regenerating enzymes.**

**
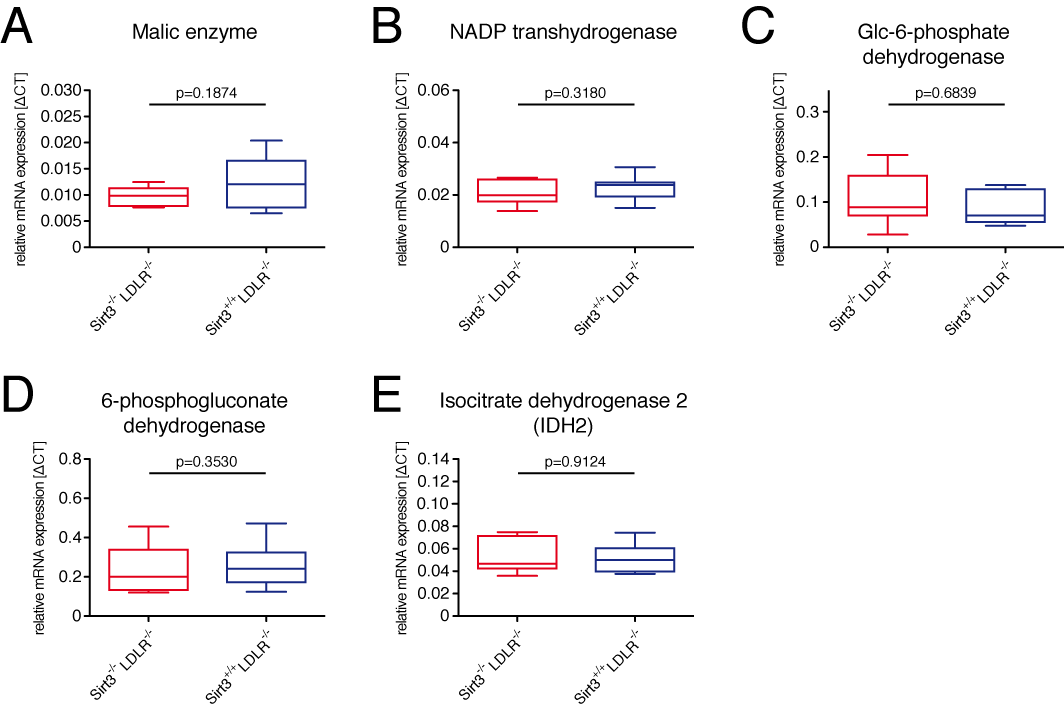
**

**Figure S2:** Eight-week old male *LDLR^-/-^* and *LDLR^-/-^ Sirt3^-/-^* mice were fed a high-cholesterol diet (1.25% w/w) for 12 weeks before mice were harvested and mRNA was isolated. Aortic expression analyses of the key NADPH regenerating enzymes were assessed using quantitative PCR. **(A)** Malic enzyme. **(B)** NADPH transhydrogenase. **(C)** Glucose-6-phosphate dehydrogenase (Glc-6-phosphate dehydrogenase). **(D)** 6-Phosphogluconate dehydrogenase. **(E)** Isocitrate dehydrogenase 2 (IDH2). Box plots show interquartile ranges, whiskers indicate minima and maxima.

**Figure S3: Sirt3 deficiency leads to hepatic global mitochondrial hyperacetylation both after high-cholesterol diet and normal chow.**

**
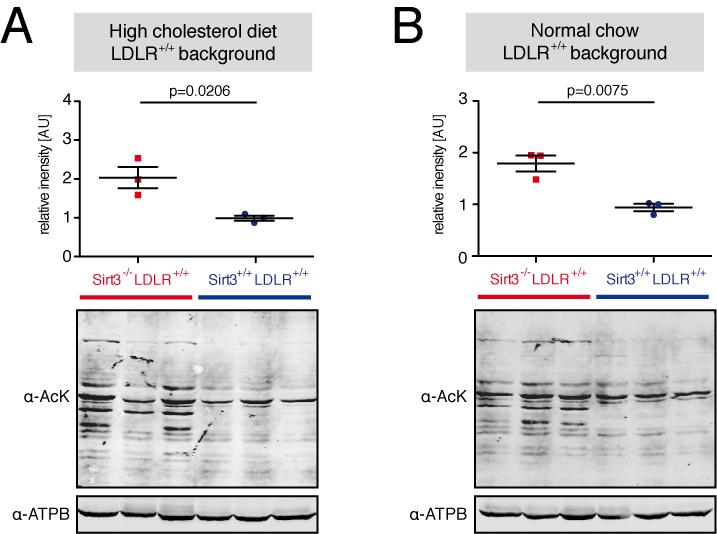
**

**
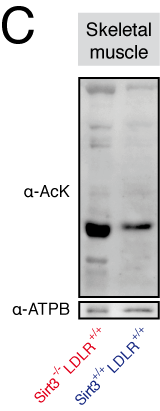
**

**Figure S3:** Eight-week old male *Sirt3^-/-^,* *Sirt3^-/-^ LDLR^-/-^,* and wiltdtype mice, respectively, were fed a high-cholesterol diet (1.25% w/w) or normal chow for 12 weeks before mice were harvested. Mitochondrial protein was isolated from livers (A & B) and gastrocnemius muscle, respectively, electrophoretically separated and probed with anti-acetyl lysine (α-AcK). **(A)** Hepatic mitochondrial protein acetylation after 12 weeks of high-cholesterol diet. **(B)** Hepatic mitochondrial protein acetylation after 12 weeks of normal chow. **(C)** Gastrocnemic mitochondrial protein acetylation after 12 weeks of high-cholesterol diet. ATP-synthase subunit ß (ATPB) served as loading control. Data are presented as means ± SEM with superimposition of individual data points.

**Figure S4:** **Loss of Sirt3 does not affect epididymal white adipose tissue, liver or spleen mass in *LDLR^-/^*^-^ mice.**


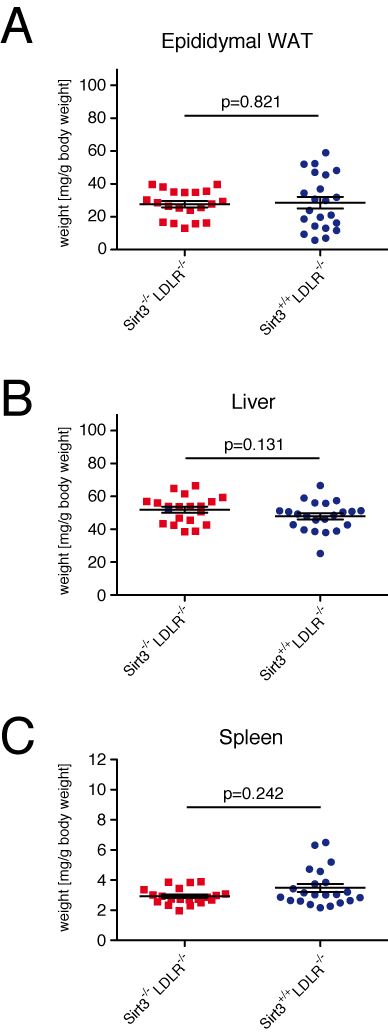


**Figure S4:** Eight-week old male *LDLR^-/-^* and *LDLR^-/-^Sirt3^-/-^* mice were fed a high-cholesterol diet (1.25% w/w) for 12 weeks before mice were harvested. **(A)** Epididymal white adipose tissue (WAT) mass. **(B)** Liver mass. **(C)** Spleen mass. Data are presented as means ± SEM with superimposition of individual data points.

**Figure S5**: **Loss of Sirt3 does not affect metabolic substrate preference or food intake.**


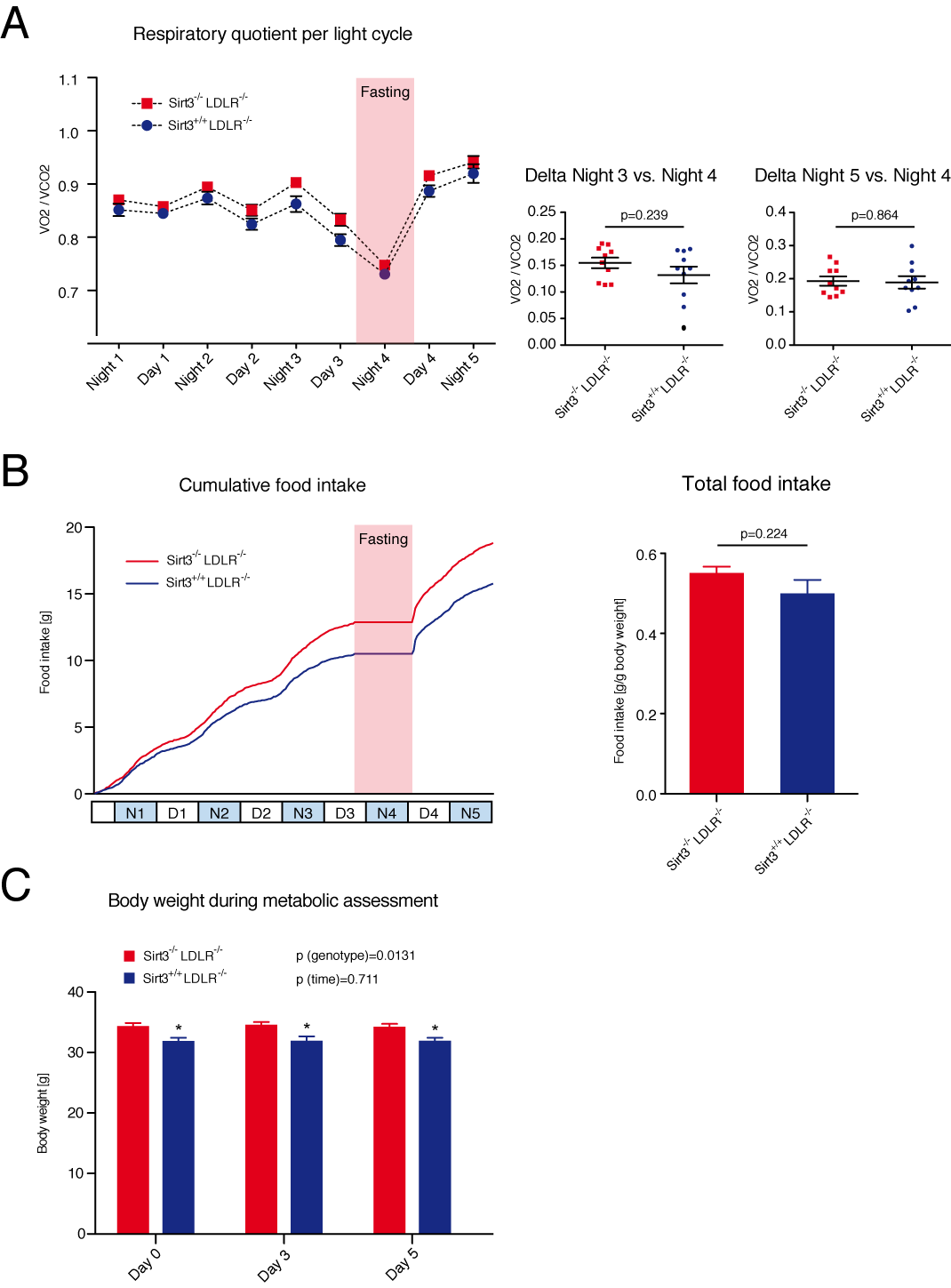


**Figure S5:** After a 12-week high-cholesterol diet (1.25% w/w) different metabolic parameters were assessed in individually-caged *LDLR^-/-^* and *LDLR^-/-^Sirt3^-/-^* mice during five light cycles. **(A)** Respiratory quotient averaged per day/night (left panel); respiratory quotient drop during fasting, determined by subtracting the individual, fed (Night 3) to fasted (Night 4) averages (center panel, « Delta N3 vs. N4 ») and respiratory quotient rebound upon refeeding, determined by subtracting refed (Night 5) to fasted (Night 4) averages (right panel, « Delta N4 vs. N5 »). **(B)** Cumulative, real-time feeding (left panel) and total feeding (right panel) over the whole experiment. **(C)** Body weights before (Day 0), during (Day 3) and after (Day 5) the experiment. Data are presented as means ± SEM, with superimposition of individual data points in « Delta » panels. N=Night, D=Day. *) p<0.05 compared with *LDLR^-/-^Sirt3^-/-^* mice.

**SUPPLEMENTARY METHODS**

**Tissue harvesting and processing**

Mice were anesthetized using isoflurane. After medial thoraco- and laparotomy the left ventricle was punctured and blood was drawn. Thereafter, the right atrium was incised and the vascular system was rinsed briefly with cold normal saline (0.9% w/v) before organs were explanted. For histological examination, tissue was embedded in OCT (optimal cutting temperature) compound (Tissue-Tek) and immediately frozen on dry ice; for biochemical analyses samples were snap frozen in liquid nitrogen. All samples were stored at -80°C until analysis.
